# The association between biochemical control and cardiovascular risk factors in acromegaly

**DOI:** 10.1186/s12902-017-0166-6

**Published:** 2017-03-09

**Authors:** John D. Carmichael, Michael S. Broder, Dasha Cherepanov, Eunice Chang, Adam Mamelak, Qayyim Said, Maureen P. Neary, Vivien Bonert

**Affiliations:** 1 Cedars-Sinai Medical Center, 8700 Beverly Blvd, Los Angeles, CA 90048 USA; 2 Partnership for Health Analytic Research, LLC, 280 S. Beverly Dr., Suite 404, Beverly Hills, CA 90212 USA; 3 Novartis Pharmaceuticals Corporation, One Health Plaza, East Hanover, NJ 07936-1080 USA

**Keywords:** Chart review, Patient registry, Acromegaly, Biochemical control, Comorbidities

## Abstract

**Background:**

The study aim was to estimate the proportion of acromegaly patients with various comorbidities and to determine if biochemical control was associated with reduced proportion of cardiovascular risk factors.

**Methods:**

Data were from a single-center acromegaly registry. Study patients were followed for ≥12 months after initial treatment. Study period was from first to last insulin-like growth factor-I and growth hormone tests.

**Results:**

Of 121 patients, 55% were female. Mean age at diagnosis was 42.4 (SD: 15.0). Mean study period was 8.8 (SD: 7.2) years. Macroadenomas were observed in 93 of 106 patients (87.7%), and microadenomas in 13 (12.3%). Initial treatment was surgery in 104 patients (86%), pharmacotherapy in 16 (13.2%), and radiation therapy in 1 (0.8%). Of 120 patients, 79 (65.8%) achieved control during the study period. New onset comorbidities (reported 6 months after study start) were uncommon (<10%). Comorbidities were typically more prevalent in uncontrolled versus controlled patients—24 (58.5%) vs. 33 (41.8%) had hypertension, 17 (41.5%) vs. 20 (25.3%) had diabetes, 11 (26.8%) vs. 16 (20.3%) had sleep apnea, and 3 (7.3%) vs. 3 (3.8%) had cardiomyopathy—except for colon polyps or cancer (19.5% vs. 20.3%), left ventricular hypertrophy (9.8% vs. 11.4%), and visual defects (14.6% vs. 17.7%).

**Conclusions:**

A greater number of comorbidities were observed in biochemically uncontrolled patients with acromegaly compared to their controlled counterparts in this single-center registry. About a third of the patients remained uncontrolled after a mean of >8 years of treatment, demonstrating the difficulty of achieving control in some patients.

## Background

Acromegaly results from excessive growth hormone (GH) production, usually caused by a benign pituitary adenoma. GH has direct metabolic effects and also stimulates hepatic insulin-like growth factor-I (IGF-1) production. IGF-1 in turn facilitates somatic growth and metabolic function [1]. The disease affects between 40 and 130 individuals per million persons, or approximately 20,000 people in the US [2], and recent studies indicate incidence of pituitary tumors in the US is increasing [3]. Although untreated acromegaly has clinically significant consequences, most signs and symptoms appear slowly, often resulting in delayed diagnosis. Most acromegaly patients are diagnosed at an average age of about 40 years [2, 4]. Diagnosis is made clinically on the basis of typical signs and symptoms and confirmed with laboratory assessment of GH and/or IGF-1 levels.

The primary goal of therapy is to normalize GH and IGF-1 levels, as these values have been shown to correspond to a reduction in mortality similar to that of an unaffected population [5, 6]. Initial treatment is generally transsphenoidal surgical resection of the adenoma, but about half of the patients require additional treatment [7]. Current guidelines recommend somatostatin receptor ligands (SRLs) (octreotide and lanreotide) or GH-receptor antagonist pegvisomant for initial medical therapy in patients with moderate-to-severe signs and symptoms of GH excess. In patients with very mild signs and symptoms, guidelines suggest an initial trial of the dopamine agonist cabergoline [7, 8].

In addition to direct effects from the tumor mass, untreated patients with acromegaly may develop diabetes mellitus, hypertension, cardiomyopathy, sleep apnea, and colon polyps at rates much higher than the non-acromegaly population [9]. The risk of developing these comorbid conditions generally increases with the length and severity of biochemical abnormalities. Successful treatment appears to reduce myocardial thickness [10] and improve sleep apnea [11], but a link between biochemical control and hypertension, diabetes, and other key outcomes has not been established. We sought, using medical records from a single referral center, to estimate the proportion of acromegaly patients with various comorbid conditions, and to determine if biochemical disease control was associated with reduced rate of cardiovascular risk factors.

## Methods

This was a prospective cohort study of an acromegaly registry established at the Cedars-Sinai Medical Center Pituitary Center (CSMC-PC), with retrospective chart data at CSMC-PC for some patients dating as far back as 1985. Consenting patients had data abstracted from medical records and entered in a database. This registry contains data on demographics, medical and surgical therapy, symptoms, laboratory values, cardiology and colonoscopy results, pathology, radiology, surgical information, and visual field data. The database is updated periodically. The current study focused on acromegaly patients treated at the center from 1985 through June 2013. Registry participants followed for at least 12 months after initial treatment were eligible for inclusion in the current study. The first and last values for IGF-1 and GH tests for each patient were used to define the study start and end dates. The study was approved by the CSMC Institutional Review Board.

Baseline measures, determined in the 6 months following the first recorded biochemical test, included patient demographics (age, sex, and race/ethnicity), tumor size, and presence of hormonal abnormalities (prolactin elevation [hyperprolactinemia], adrenal insufficiency [use of adrenal replacement], gonadal insufficiency [use of sex steroid replacement], and hypothyroidism [use of thyroid replacement]). Adrenal insufficiency was diagnosed with standard cortrosyn (ACTH) stimulation testing with a cutoff point of 18 mcg/dl. Treatments for acromegaly, including surgery, radiation, and pharmacotherapy, were recorded, as were the dates of those treatments. Patients receiving presurgical pharmacotherapy of short duration were reported simply as having had surgery, unless pharmacotherapy was continued after the procedure. The presence of comorbid conditions, including hypertension (i.e., diagnosis of hypertension or use of hypertensive medication), diabetes (i.e., diagnosis of diabetes or HbA1c ≥6.5% or use of antihyperglycemic medication), left ventricular hypertrophy (LVH), cardiomyopathy, congestive heart failure (CHF), sleep apnea, colon polyps, colon cancer, and visual field defects (i.e., diagnosis of any visual acuity impairment or visual field cut), was recorded, as was the use of antihypertensive and antihyperglycemic medications. All comorbidities were diagnosed by specialists in their respective fields, using standard diagnostic methods. All GH and IGF-1 values were recorded. The primary predictor variable was biochemical control at study end, defined as a last IGF-1 less than or equal to the upper limit of normal for patient’s age and gender [12, 13]. Considering the possiblity of discordance between values of GH and IGF-I in different treatment scenarios, to maintain a robust definition of control we opted to rely solely on IGF-I for this analysis [13]. The primary and secondary outcomes of interest were the proportion of patients with new onset (noted any time after the first 6 months of observation) comorbidities and the proportion with a comorbidity of interest at any time during the study period.

Descriptive statistics, including means, standard deviations (SD), medians, and percentages, were estimated for all study measures as applicable, and reported separately for patients who did and did not achieve biochemical control. For the primary analysis, presence of existing and new onset comorbidities were examined in patients, stratified on the basis of whether or not they had attained biochemical control. All data transformations and statistical analyses were performed using SAS® version 9.4 (SAS Institute, Cary, NC).

## Results

A total of 121 acromegaly patients met the inclusion criteria, provided written consent, and were included in the study. The mean (SD) patient age at the time of this study was 55.4 (16.7) years, and the mean age at diagnosis was 42.4 (15.0). Overall, 67 (55%) patients were female. Race/ethnicity was reported as Caucasians for 88 (72.7%) patients, Asian for 16 (13.2%), and Hispanic for 12 (9.9%); 5 (4.1%) patients were of other race/ethnicity. The mean (SD) time from first to last recorded biochemical value (study period) was 8.8 (7.2) years, and the median (25th to 75th percentile) was 5.8 (3.2-13.7) years. For 106 patients, data were available on initial tumor size. Macroadenomas were observed in 93 patients (87.7%) and microadenomas in 13 patients (12.3%) in this cohort. On presentation, 20 (16.5%) patients had gonadal insufficiency, 19 (15.7%) had hypothyroidism, 18 (14.9%) had adrenal insufficiency, and 1 (0.8%) had elevated prolactin (Table 1).

**Table 1:** Baseline characteristics of 121 acromegaly patients

		All *N* = 121
Age, year	Mean	55.4
	(SD)	(16.7)
Age^a^ at diagnosis, year	n	109
	Mean	42.4
	(SD)	(15.0)
Female	n	67
	(%)	(55.4)
Race/ethnicity		
Caucasian	n	88
	(%)	(72.7)
Asian	n	16
	(%)	(13.2)
Hispanic	n	12
	(%)	(9.9)
Other	n	5
	(%)	(4.1)
Years of follow-up	Mean	8.8
	(SD)	(7.2)
	25^th^ percentile	3.2
	Median	5.8
	75^th^ percentile	13.7
Tumor size^b^	n	106
Macroadenoma	n	93
	(%)	(87.7)
Microadenoma	n	13
	(%)	(12.3)
Additional Hormonal Abnormalities		
Gonadal insufficiency	n	20
	(%)	(16.5)
Hypothyroidism	n	19
	(%)	(15.7)
Adrenal insufficiency	n	18
	(%)	(14.9)
Prolactin elevation	n	1
	(%)	(0.8)

^a^109 patients had information about age at diagnosis

^b^106 patients had tumor size information

Initial treatment was surgery, observed in 104 patients (86%), which included 13 patients with short duration pre-surgical pharmacologic therapy. Initial treatment was pharmacologic in 16 patients (13.2%), and radiation therapy in the 1 remaining patient. For patients treated with primary medical therapy, somatostatin analogues were the most common initial pharmacologic treatment, observed in 11 patients (9.1%), followed by dopamine agonists in 5 patients (4.1%) (Table 2). By the end of a median of 5.8 years of follow-up, 92 (76%) patients had been treated with multiple modalities. Of 104 initially surgically treated patients, 78 (75%) required second line treatment: 67 had pharmacotherapy, 7 a subsequent surgery, and 4 received radiotherapy as second line treatment.

**Table 2:** Initial treatment

		All *N* = 121
Pituitary surgery^a^	n	104^b^
	(%)	(86.0)
Radiation	n	1
	(%)	(0.8)
Pharmacotherapy	n	16
	(%)	(13.2)
Somatostatin analogues	n	11
	(%)	(9.1)
Dopamine agonists	n	5
	(%)	(4.1)

^a^Includes patients with pre-surgical medication (≤6 months medication prior to surgery)

^b^13 had presurgical medication

Of the 121 subjects, 1 had no IGF-1 values reported, and consequently was dropped from the main analysis. Among the remaining 120, 79 (65.8%) had achieved biochemical control during the period of observation (mean 8.8 years, median 6.1 years) and 41 (34.2%) had not. The mean IGF-1 level at study start was 260% of upper limit of normal (ULN) in patients who were eventually controlled and 242% of ULN in those not controlled. The mean last IGF-1 was 67.7% of ULN in controlled and 177.8% in uncontrolled patients (Table 3).

**Table 3:** Change from initial to last IGF-1 over the study period

		Controlled *N* = 79; 65.8%	Uncontrolled *N* = 41; 34.2%	All *N* =120
Baseline IGF-1 (% of UNL)	Mean	260.3	241.6	253.9
Last IGF-1 (% of UNL)	Mean	67.7	177.8	105.3
Difference (last test value minus the baseline value)	Mean	192.6	−63.8	−148.6

*IGF-1* insulin-like growth factor I, *ULN* upper limit of normal

New onset comorbidities (those first reported after the initial six months of the study period) were uncommon. There were 6 (7.6%) new cases of hypertension and 3 (3.8%) of diabetes in controlled patients compared to 3 (7.3%) and 4 (9.8%) in uncontrolled patients. There were 7 (8.9%) cases of LVH in controlled compared to 2 (4.9%) in uncontrolled patients. No new cases of cardiomyopathy were observed during the study period. There was 1 case of colon polyp or cancer in each group (1.3% controlled vs. 2.4% uncontrolled), 6 (7.6%) cases of visual field defects in controlled and 2 (4.9%) cases in uncontrolled patients, with visual field defects present in all at presentation, and not worsening during therapy (Table 4).

**Table 4:** New onset comorbid conditions^a^, stratified by last observed biochemical control

		Controlled *N* = 79; 65.8%	Uncontrolled *N* = 41; 34.2%	All *N* =120
Cardiovascular				
Hypertension	n	6	3	9
	(%)	(7.6)	(7.3)	(7.5)
Diabetes mellitus	n	3	4	7
	(%)	(3.8)	(9.8)	(5.8)
Left ventricular hypertrophy	n	7	2	9
	(%)	(8.9)	(4.9)	(7.5)
Cardiomyopathy or heart failure	n	0	0	0
	(%)	(0.0)	(0.0)	(0.0)
Other				
Sleep apnea	n	0	0	0
	(%)	(0.0)	(0.0)	(0.0)
Colonic polyps or colon cancer	n	1	1	2
	(%)	(1.3)	(2.4)	(1.7)
Visual field defects	n	6	2	8
	(%)	(7.6)	(4.9)	(6.7)

^a^New onset comorbidities were defined as evidence of condition during the study period, excluding the first 6 months comprising the baseline period

Considering comorbidities identified at any time during care, rather than new onset comorbidities, in the controlled group, 33 (41.8%) had hypertension compared to 24 (58.5%) in the uncontrolled group. Diabetes was observed in 20 (25.3%) of controlled compared to 17 (41.5%) of uncontrolled patients. LVH and cardiomyopathy were identified in 9 (11.4%) and 3 (3.8%) of controlled patients compared to 4 (9.8%) and 3 (7.3%) of uncontrolled patients. Sleep apnea was present in 16 (20.3%) of controlled and 11 (26.8%) of uncontrolled patients. Colon polyps or cancer were present in 16 (20.3%) of controlled and 8 (19.5%) of uncontrolled patients. Visual field defects were observed in 14 (17.7%) of controlled and 6 (14.6%) of uncontrolled patients (Table 5).

**Table 5:** Comorbid conditions, stratified by last observed biochemical control

		Controlled *N* = 79; 65.8%	Uncontrolled *N* = 41; 34.2%	All *N* =120
Cardiovascular				
Hypertension	n	33	24	57
	(%)	(41.8)	(58.5)	(47.5)
Diabetes mellitus	n	20	17	37
	(%)	(25.3)	(41.5)	(30.8)
Left ventricular hypertrophy	n	9	4	13
	(%)	(11.4)	(9.8)	(10.8)
Cardiomyopathy or heart failure	n	3	3	6
	(%)	(3.8)	(7.3)	(5.0)
Other				
Sleep apnea	n	16	11	27
	(%)	(20.3)	(26.8)	(22.5)
Colonic polyps or colon cancer	n	16	8	24
	(%)	(20.3)	(19.5)	(20.0)
Visual field defects	n	14	6	20
	(%)	(17.7)	(14.6)	(16.7)

## Discussion

In a large sample of acromegaly patients treated at a single US referral center, we demonstrated a greater number of cardiovascular and other comorbidities in biochemically uncontrolled patients with acromegaly compared to their controlled counterparts. We also observed that despite the use of multiple treatment modalities by experienced clinicians, about a third of the patients presenting to a tertiary referral center remained biochemically uncontrolled after a mean of more than eight years of treatment, demonstrating the difficulty of achieving biochemical control in some patients. Finally, we identified a higher prevalence of hypertension, diabetes, and sleep apnea than has been reported in other acromegaly registries [14, 15].

Cardiovascular disease in general, and LVH in particular, are a significant cause of mortality in acromegaly [1, 5]. The biochemical basis of cardiac remodeling and subsequent LVH in acromegaly is not fully established but appears to result, at least in part, as a direct effect of elevated levels of GH and IGF-1 on cardiac cells [16]. Treatment of these biochemical abnormalities improves LVH. A review of 15 studies, most less than 1 year in duration, showed consistent reduction in LVH after biochemical control of the disease [9], and subsequent studies have had similar results [10]. Factors other than a direct hormonal effect on cardiac muscle may influence development of cardiac disease. Colao et al. [17] reported that hypertension and diabetes also correlate with the presence of cardiomyopathy.

Hypertension (47.5%) and diabetes (30.8%) were the most commonly observed comorbidities during the study period, and both were more common in uncontrolled patients than in controlled patients. LVH was reported in 10.8%, and CHF in 5% of patients. Hypertension was more commonly encountered in the current study than has been reported in several European registries, and somewhat higher than a commonly cited figure of 40% [18]. For example, in a Belgian acromegaly registry of 418 patients, Bex et al. [14] reported that 39.4% of reported cases had hypertension. From a Spanish registry of more than 1000 patients, Mestron et al. [15] reported 39.1% with hypertension. Diabetes was less common (25.3%) in the Belgian study and slightly more common (36.5%) in the Spanish study [14, 15]. Hypertension and diabetes are associated with mortality in the non-acromegaly population, although the current literature is inconsistent with regard to the impact of biochemical acromegaly control on reduction in these risk factors [9]. We observed higher prevalence of both conditions in uncontrolled compared to controlled patients. New onset of major comorbidities was uncommonly encountered, regardless of biochemical control status. Hypertension (7.5%), left ventricular hypertrophy (7.5%), visual field defects (6.7%), and diabetes (5.8%) were the most frequently observed new-onset comorbidities; all other comorbidities of interest occurred were uncommon (<2%).

Despite the availability of multiple therapies, including newer pharmacologic treatments, reducing serum IGF-1 levels to within the normal range remains challenging in some patients. Overall, IGF-1 dropped substantially: from 254% of ULN at study start to 105% of ULN at study end. Achieving this reduction required the use of multiple therapeutic modalities, and even with these treatments, 34% of patients did not attain biochemical control at the end of the study. However, even among uncontrolled patients, IGF-1 values fell from 242% to 178% of ULN, suggesting that continued treatment over time is likely to provide some biochemical improvement. It is unclear with this analysis if this improvement imparts benefit in terms of comorbidities, in patients not meeting a definition of biochemical control. Non-adherence may have reduced the number of patients who were in control at the end of the study. It is unclear from the current data the extent to which patients were adherent to therapy.

This study has several limitations. Firstly, despite the relatively large sample size for a study of acromegaly, small numbers moderate the power of statistical comparisons. Secondly, the results reflect care at a single institution over more than two decades. Practices have changed significantly over that time, and the results, therefore, may not be representative of what would be experienced were the study to be repeated today. Thirdly, some patients were treated elsewhere before referral to CSMC-PC. This is a study of population of patients with complex acromegaly, referred for specialized tertiary or quaternary care. Data entered in the registry reflects care from a variety of providers both within and outside CSMC and documentation may not have been optimal. Finally, this is not a randomized-placebo controlled study, where the study design would have required strict medication titration protocol and oversight until control was attained. This is a registry where there could have been treatment interruption (e.g., insurance coverage) and unsupervised or possibly sub-optimal dose titration that may have had impact attainment of control.

## Conclusion

Overall, a greater number of comorbidities were observed in biochemically uncontrolled patients with acromegaly compared to their controlled counterparts in this single-center registry. This study indicates that about a third of the patients remained uncontrolled after a mean of >8 years of treatment, demonstrating the difficulty of achieving control in some patients.

## Abbreviations

CHF: Congestive heart failure
CSMC-PC: Cedars-Sinai Medical Center Pituitary Center
GH: Growth hormone
IGF-1: Insulin-like growth factor-I
LVH: Left ventricular hypertrophy
SD: Standard deviations
SRLs: Somatostatin receptor ligands
ULN: Upper limit of normal
